# A Dual-Circular RNA Signature as a Non-invasive Diagnostic Biomarker for Gastric Cancer

**DOI:** 10.3389/fonc.2020.00184

**Published:** 2020-02-21

**Authors:** Li Han, Xiaoying Zhang, Aimin Wang, Yang Ji, Xuelei Cao, Qiaoji Qin, Tao Yu, Huan Huang, Lei Yin

**Affiliations:** ^1^School of Nursing, Qingdao University, Qingdao, China; ^2^Department of Cardiology, The Affiliated Hospital of Qingdao University, Qingdao, China; ^3^Department of Emergency Internal Medicine, The Affiliated Hospital of Qingdao University, Qingdao, China

**Keywords:** gastric cancer, circRNAs, plasma, diagnosis, signature, Hsa_circ_0020187, Hsa_circ_0005051

## Abstract

Gastric cancer (GC) is the TOP3 leading cause of human mortality in malignant tumors. Notwithstanding, the association between GC and circRNAs is not clear. The purpose of this research was to determine the association between GC progression and circRNAs. The data of circRNAs was obtained from the Gene Expression Omnibus (GEO) database to identify gene, which differentially expressed circRNAs in GC tissues and paired normal tissues. The expression of circRNAs in cancer tissues and normal tissues were tested, and the target circRNA was verified before and after surgery in the plasma. A circRNA-micro(mi)RNA-mRNA competing endogenous RNAs (ceRNAs) network was established, and GO and KEGG analysis are performed. Five candidate circRNAs were identified through bioinformatics analysis. Hsa_circ_0021087 and hsa_circ_0005051 were both downregulated in GC tissues, cells and plasma by RTq-PCR. Additionally, there was a significant difference in the expression of plasma hsa_circ_0021087 in patients with GC at the preoperative and postoperative stages (*P* < 0.001). Hsa_circ_0021087 also promoted the proliferation of GC cells *in vitro*. Next, the circRNA-miRNA-mRNA network of hsa_circ_0021087 was predicted, which may be associated with the development of GC by bioinformatics analysis. In summary, the aforementioned dual-circular RNAs may have important implications on the potential, novel and non-invasive diagnostic method for patients with GC.

## Introduction

Gastric cancer (GC) has become the third leading cause of cancer-associated death and is the 6th common cancer in the world (1). Although much progress has been made in the diagnosis of GC and survival after radiotherapy, chemotherapy and surgery, the 5-year survival rate in most countries is still <30% (2). Hence, in order to improve the early diagnosis, treatment and prevention of GC, the discovery of new molecular biomarkers and therapeutic targets is significant.

Circular RNAs (circRNAs), which is a novel class of non-coding RNAs, have a covalently closed loop, derived by pre-mRNA back-splicing of genes (3). CircRNAs usually express tissues/developmental stage-specificity and most are evolutionarily conserved in different organisms, including fruit flies, mammals and plants (4). With the discovery of more and more circRNAs, the function of circRNAs has been gradually reported, especially to promote the physiological and pathological progression of disease, such as cell proliferation, apoptosis, migration and carcinogenesis (5). In the past few years, studies have demonstrated that circRNAs are involved in many malignancies, such as gastric cancer (6), hepatocellular carcinoma (7), colon cancer (8), and pancreatic carcinoma (9), by acting as miRNA sponges and inhibiting target gene expression.

Furthermore, circRNAs could act as new signatures because of their conservatism, specificity, and stability (3, 10). In addition, circRNA can exist in exosomes and even the blood (11, 12). Liquid biopsies are more convenient and less invasive than traditional biopsies, and can be used to analyze biomarkers in specific tumor tissues. Therefore, circulating tumor circRNAs (ct circRNAs) can be applied as potential signatures for the diagnosis of cancer. Tang et al. (13) had demonstrated that plasma circ-KIAA1244 might be a potential signature in patients with GC. As far as we know, however, there are few studies on circRNAs in the plasma of patients with GC.

The aim of this research was the identification and verification of specific circRNAs in the blood for the diagnosis of early GC. Bioinformatics was used to analyze differentially expressed circRNAs and RTq-PCR (reverse-transcription-quantitative) was used to verify the aforementioned circRNAs. The expression of circRNAs in cells and blood was verified at the same time. One of the most important components of competitive endogenous RNAs (ceRNAs) was demonstrated to be circRNAs (14). Micro (mi)RNAs can be bind to their binding sites through miRNA sponge, which regulates the miRNA of the target gene (3). Therefore, in order to predict the hypothetical mechanism and functions of circRNA in GC, a ceRNA network was established and its function was analyzed.

## Methods

### Clinical Samples

Tissues and peripheral blood of 70 patients were obtained from the Department of general surgery of the Affiliated Hospital of Qingdao University between March and June 2018. GC tissues and their matching paracancerous tissues were obtained from 70 patients undergoing surgery without radiotherapy or chemotherapy. All collected tissues were frozen in a −80°C refrigerator until further use. Matched blood samples were collected from 70 patients before operation and 6 days after operation. The 70 normal controls without any history of cancer were matched with GC cases by sex and age. The clinical information of samples is summarized in Table S1. The Institutional Review Board of Affiliated Hospital of Qingdao University approved this study.

### Candidate CircRNAs

CircRNA expression profiles of GC were obtained from GEO (Gene Expression Omnibus) database at https://www.ncbi.nlm.nih.gov/geo/, including GSE83521, GSE89143, and GSE93541. The Limma package was used to analyze the differently expressed circRNAs (DEcircRNAs), for which the cutoff standard was |logFC| >1 and *P* < 0.05 (15). Five circRNAs were selected from the intersection of DEcircRNAs of the aforementioned three datasets. The CircBank (http://www.circbank.cn/) was used to find the original gene associated with the circRNA (16). Furthermore, the expression levels of original genes were analyzed by the Kaplan–Meier plotter (http://kmplot.com/analysis/index.php?p=service&cancer=gastric) to evaluate their prognostic value (17).

### Cell Culture

Normal human gastric epithelium cell (GES1), MGC-803, and BGC-823 cell lines were obtained from Institutes for Biological Sciences. These cell lines were cultured in Dulbecco modified Eagle medium (DMEM) containing 10% fetal bovine serum (FBS) and grew at 5% CO_2_ of 37°C. Lipofectamine® 3,000 reagent (Invitrogen; Thermo Fisher Scientific, Inc.) was used to transfect cells with siRNA, as following the manufacturer's instructions.

### RNA Isolation and RT-qPCR

Total RNA was extracted by TRIzol reagent (Invitrogen; Thermo Fisher Scientific, Inc.) from tissues and blood. After dissolution, NanoDrop Lite spectrophotometer was used to evaluate the concentration and purity of total RNA. Subsequently, the total RNA was reverse transcribed for the synthesis of cDNA and used to conduct qPCR assays for circRNAs. GAPDH was used as the internal control, and all procedures were repeated three times. Fold change (2^−ΔΔCT^) was used to represent relative gene expression levels. Table S2 lists all primer sequences and small interference sequences, and the divergent primers designed for each circRNA.

### Cell Proliferation Assay

The CCK-8 (Cell Counting Kit-8) assay was performed to examine whether hsa_circ_0020187 was associated with cell proliferation. MGC-803 and BGC-823 cells, after transfection and culture for a day, were seeded into 96–well plates (5 × 10^3^ cells for each well). Each group was replicated in three independent wells. Cell proliferation was determined at 0, 24, 48, and 72 h by optical density (OD) at 450 nm using a spectrophotometer.

### Wound Healing Assay

The transfected cells were inoculated into a six-well plate and were allowed to reach 80% confluence. The single-cell layer was scratched with the tip of a 10-μl pipette and then washed with 1X PBS three times to clear the cell debris, and replaced with fresh serum-containing medium. The wound was allowed to heal for 24 h, and the images (Nikon Corporation) were obtained at 0 and 24 h, respectively. The wound width was calculated by ImageJ software (version 1.8.0).

### Migration Assays

Adding 10% FBS to the bottom chamber as a chemotactic agent. Approximately 5 × 10^4^ transfecting cells were added to 200 μl serum-free medium and inoculated in the upper chamber, at 5% CO_2_ of 37°C. Following 16 h, the cells in the upper chamber were gently wiped with cotton swabs. The contralateral cells of the filter were fixed with 70% ethanol for 30 min, and then stained with 0.1% crystal violet for 10 min. The images were captured under a microscope (Nikon Corporation) and the migrated cells were counted by ImageJ software.

### Construction of ceRNA Network and Functional Analysis

CircInteractome (18) and circBank (16) were used to predict the miRNAs of candidate circRNAs. Furthermore, the Starbase (19) and TargetScan (20) were applied to predict the miRNA-target. The circRNA-miRNA-mRNA network was calculated and constructed by GDCRNATools packages and visualized with Cytoscape (21). In addition, in order to further understand the function of ceRNAs, GO and KEGG pathway analyses were performed through the clusterProfiler (22).

### Statistical Analysis

All the data were analyzed by GraphPad Prism v7.0, R software 3.6.1 and the SPSS v23.0. The paired *t*-test was used to evaluate the differences in the circRNA expression levels before and after operating groups. The associations between circRNA expression in patients and the clinicopathological factors with GC were analyzed by Chi-square test. The diagnostic value of these specific circRNAs was assessed by ROC (receiver operating characteristic) curve analysis and the AUC (area under the ROC curve). *P* < 0.05 was considered statistically significant.

## Results

### Identification of CircRNAs

**Figure 1:**
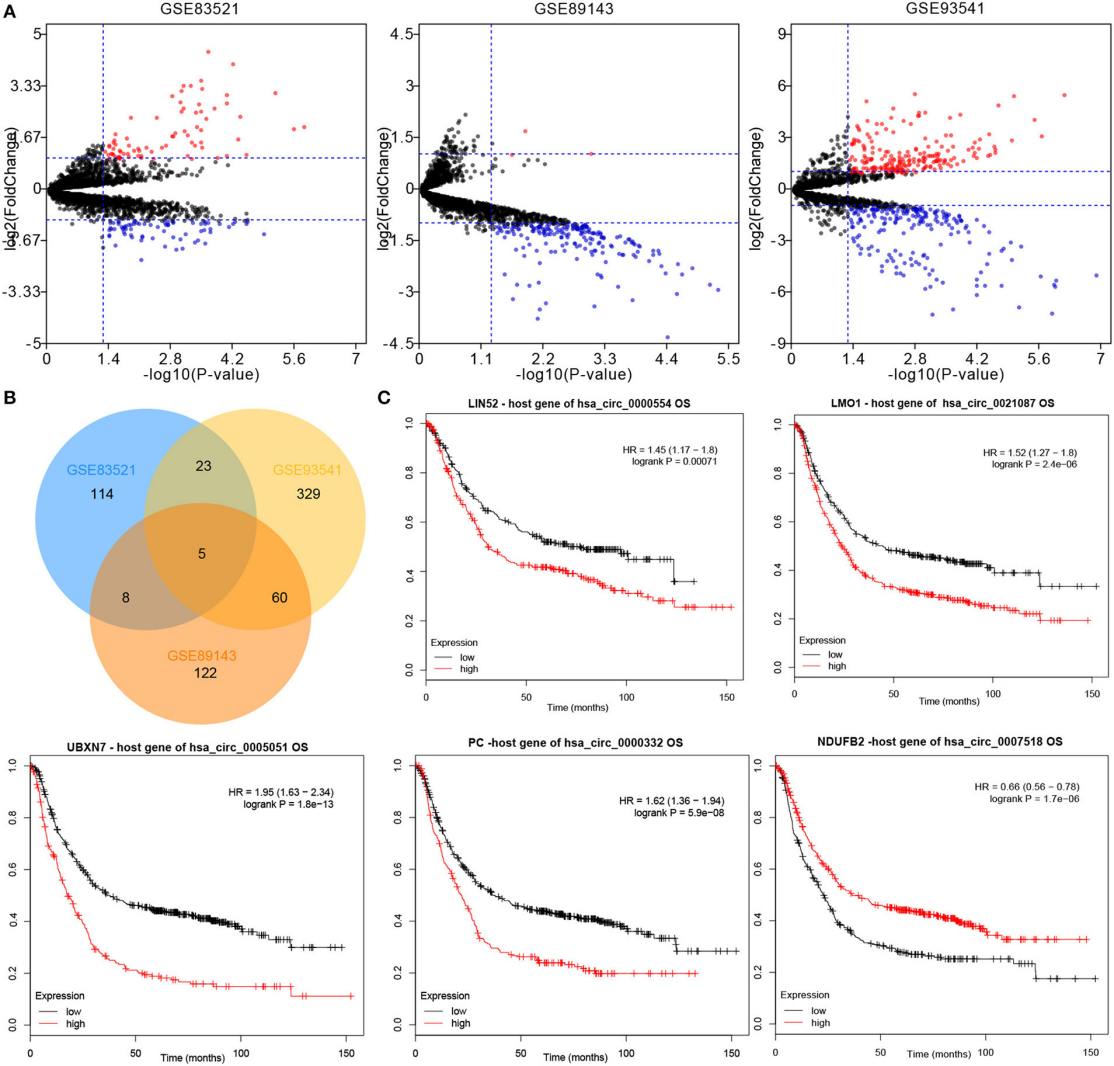
CircRNA expression profiles of GC and matched normal tissues. **(A)** Differentially expressed circRNAs between the tumor and non-tumor groups were shown by volcano plots. **(B)** Intersection of differentially expressed circRNAs in the datasets: GSE83521, GSE89143, and GSE93541. **(C)** The overall survival of five circRNAs host genes in GC patients was analyzed by Kaplan–Meier plotter. Log-rank test was used to determine the statistical significance. CircRNA, circular RNA; GC, gastric cancer.

By using the Limma package, the datasets GSE83521, GSE89143, and GSE93541 from the GEO database were re-analyzed, which identified DEcircRNAs in GC tissues and paired non-tumor tissues (Figure 1A). Following the intersection, a total of five downregulated circRNAs were obtained (hsa_circ_0000554, hsa_circ_0021087, hsa_circ_0005051, hsa_circ_0000332, and hsa_circ_0007518) (Figure 1B). Subsequently, in order to identify the above candidate circRNAs, the prognostic value of their host gene in gastric samples were analyzed using the Kaplan–Meier plotter (Figure 1C). The potential prognostic value of the aforementioned five circRNAs was demonstrated.

### Verifying the Expression Levels of circRNAs

**Figure 2:**
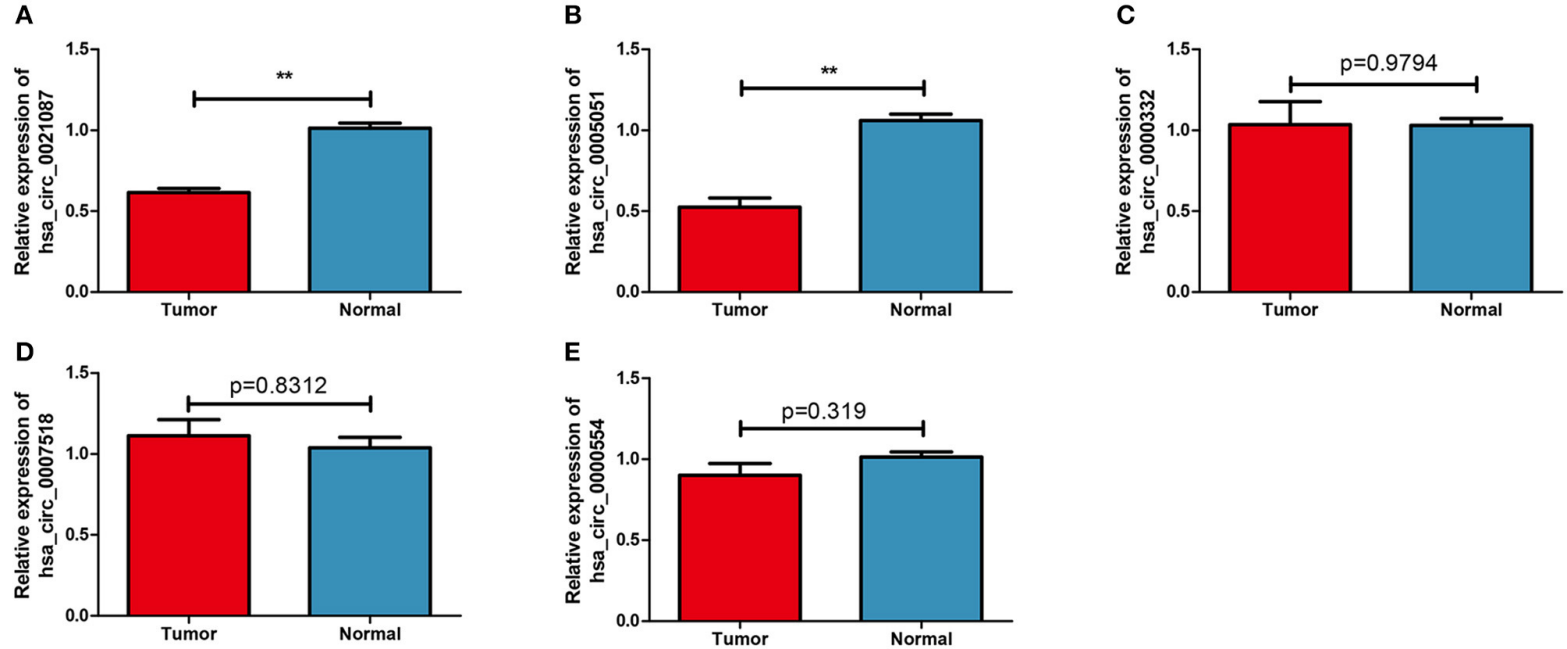
Candidate circular RNAs in patients with GC and normal groups were analyzed by reverse transcription-quantitative PCR. **(A)** Compared with the normal group, the expression of plasma Hsa_circ_0021087 in patients with GC was down-regulated. **(B)** Hsa_circ_0005051 was downregulated in GC patients. Hsa_circ_0000332 **(C)**, hsa_circ_0007518 **(D)**, and hsa_circ_0000554 **(E)** were determined with no differential expression between the two groups. **P < 0.01. GC, gastric cancer.

The expression levels of five candidate circRNAs were verified by RTq-PCR in 70 GC samples and 19 normal blood samples. The results indicated that both hsa_circ_0021087 and hsa_circ_0005051 were downregulated in GC (*P* < 0.01; Figures 2A,B), which was consistent with the results of the microarray analysis. Nonetheless, the expression levels of hsa_circ_0000332, hsa_circ_0007518 and hsa_circ_0000554 showed no significant difference in the plasma of patients with GC compared with the healthy controls (*P* = 0.9794, Figure 2C; *P* = 0.8312, Figure 2D; *P* = 0.319, Figure 2E). The above two circRNAs sources are shown in Figure S1.

### Hsa_circ_0021087 and hsa_circ_0005051 Diagnostic Value in Patients With GC

**Figure 3:**
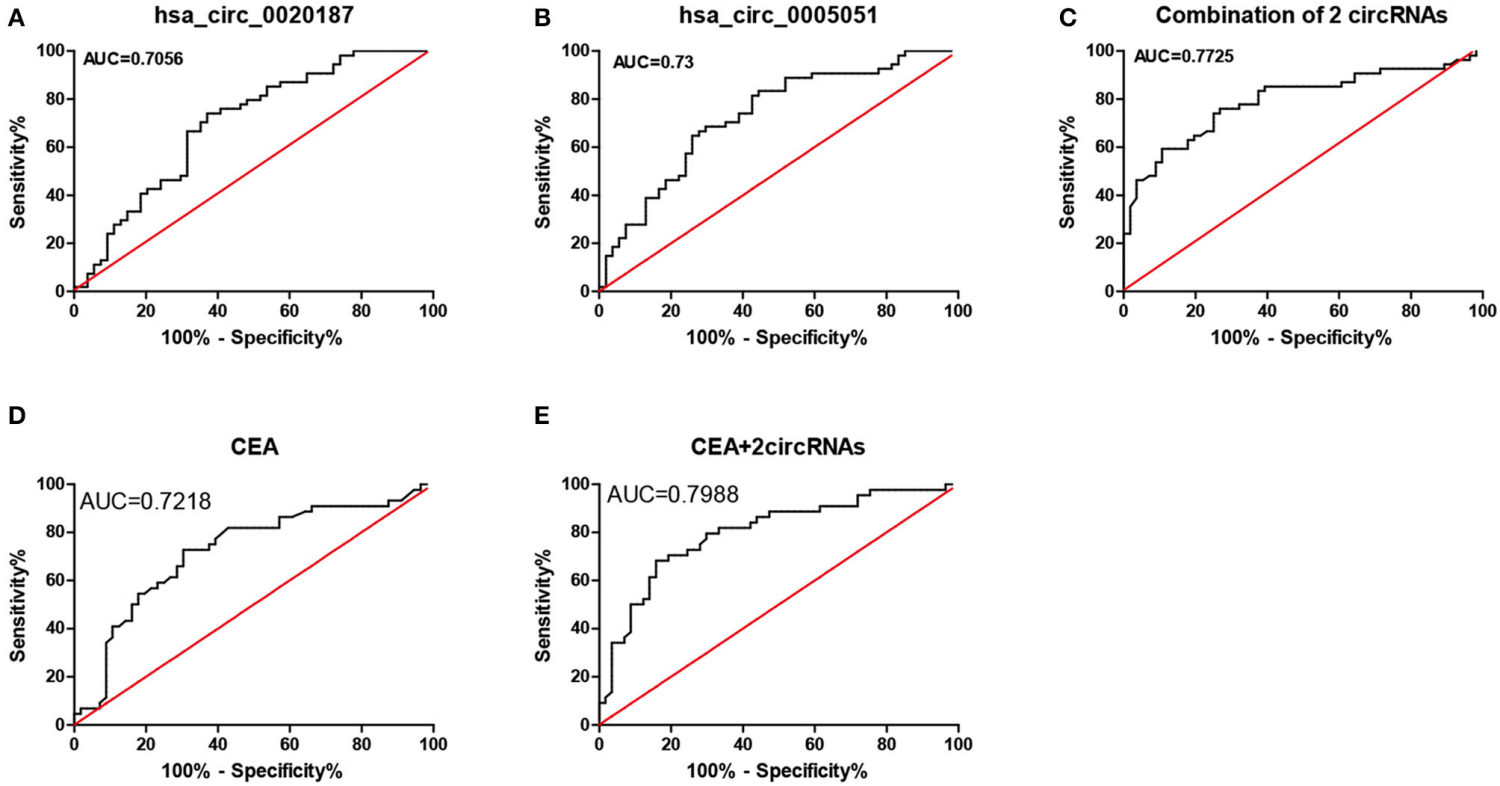
ROC analysis of hsa_circ_0021087, hsa_circ_0005051, and the combination of the two molecular biomarkers were used in the diagnosis of GC. **(A)** The hsa_circ_0021087 area under the ROC curve (AUC) 0.7056, *P* < 0.001. **(B)** Hsa_circ_0005051: 0.73, *P* < 0.001. **(C)** Combination of 2 circRNAs: 0.7725, *P* < 0.001. **(D)** CEA: 0.7218, *P* < 0.001. **(E)** CEA and 2 circular RNAs: 0.7988, *P* < 0.001. ROC, receiver operating characteristic; AUC, area under the curve; CEA, carcinoembryonic antigen.

The diagnostic value of hsa_circ_0021087 and hsa_circ_0005051 from the Figure 3 shows the potential of ROC analysis and CEA to distinguish GC patients from normal controls. Hsa_circ_0021087 and hsa_circ_0005051's AUC was 0.7056 and 0.73, respectively. The expression values of the above dual-circRNA signature and CEA combination that provide the best distinction was analyzed, which resulted in an AUC of 0.7988, *P* < 0.0001.

### Association Between the Clinicopathological Characteristics and the Expression Levels of the Dual Circular RNAs

The correlation between the clinical characteristics of patients with GC and the expression levels of the two kinds of circRNAs was performed to evaluate by additional analysis. As shown in Table 1, hsa_circ_0021087 and hsa_circ_0005051 expression was associated with tumor size and TNM stage, respectively (*P* = 0.017; *P* = 0.032). However, the results showed no association between circRNAs and the patients' age, sex, and lymph node metastasis.

**Table 1:** Correlations between hsa_circ_00021087 and hsa_circ_0005051 expression and clinical characteristics in GC patients.

**Characteristic**	**Case**	**hsa_circ_00021087**			**hsa_circ_0005051**		
		**Low**	**High**	***p*-value**	**Low**	**High**	***p*-value**
All cases	70	54	16		49	21	
Age (yeas)				0.453			0.834
<65	32	26	6		22	10	
≥65	38	28	10		27	11	
Gender				0.285			0.333
Female	27	19	8		20	7	
Male	43	35	8		29	14	
Tumor size (cm)				0.017^*^			0.292
<5	30	19	11		19	11	
≥5	40	35	5		30	10	
Lymph node metastasis				0.674			0.177
Negative	19	14	5		11	8	
Positive	51	40	11		38	13	
TNM stage				0.794			0.032^*^
I–II	33	25	8		19	14	
III–IV	37	29	8		30	7	

* *p < 0.05*.

### Hsa_circ_0021087 Displayed Differential Expression of GC Patients' Preoperative and Postoperative Stages in Blood

**Figure 4:**
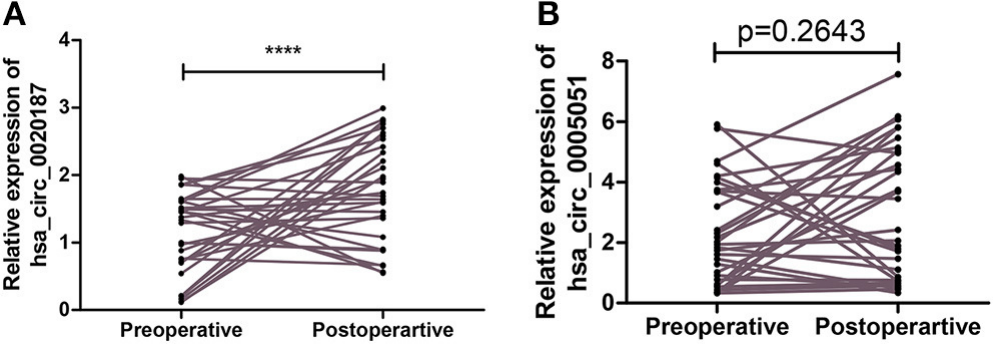
Comparison the expression of hsa_circ_0021087 and hsa_circ_0005051 in patients' plasma with gastric cancer before and after operation. **(A)** Downregulated hsa_circ_0021087 was detected at a higher level in plasma samples after surgical resection. **(B)** Hsa_circ_0005051 was detected at preoperative and postoperative stages with no significant differential expression. ****P < 0.0001.

The expression of hsa_circ_0021087 and hsa_circ_0005051 was detected from the blood of patients with GC before and after operation. The findings revealed that the expression of hsa_circ_0021087 was significantly increased in 24 of the 31 (77.42%) patients after surgery (*P* < 0.001; Figure 4A). However, it was found that no obvious sense in expression of hsa_circ_0005051 was discovered before and after operation (*P* = 0.11; Figure 4B).

### Hsa_circ_0021087 Exerts Oncogenic Effects on the MGC-803 and BGC-823 Cell Lines

**Figure 5:**
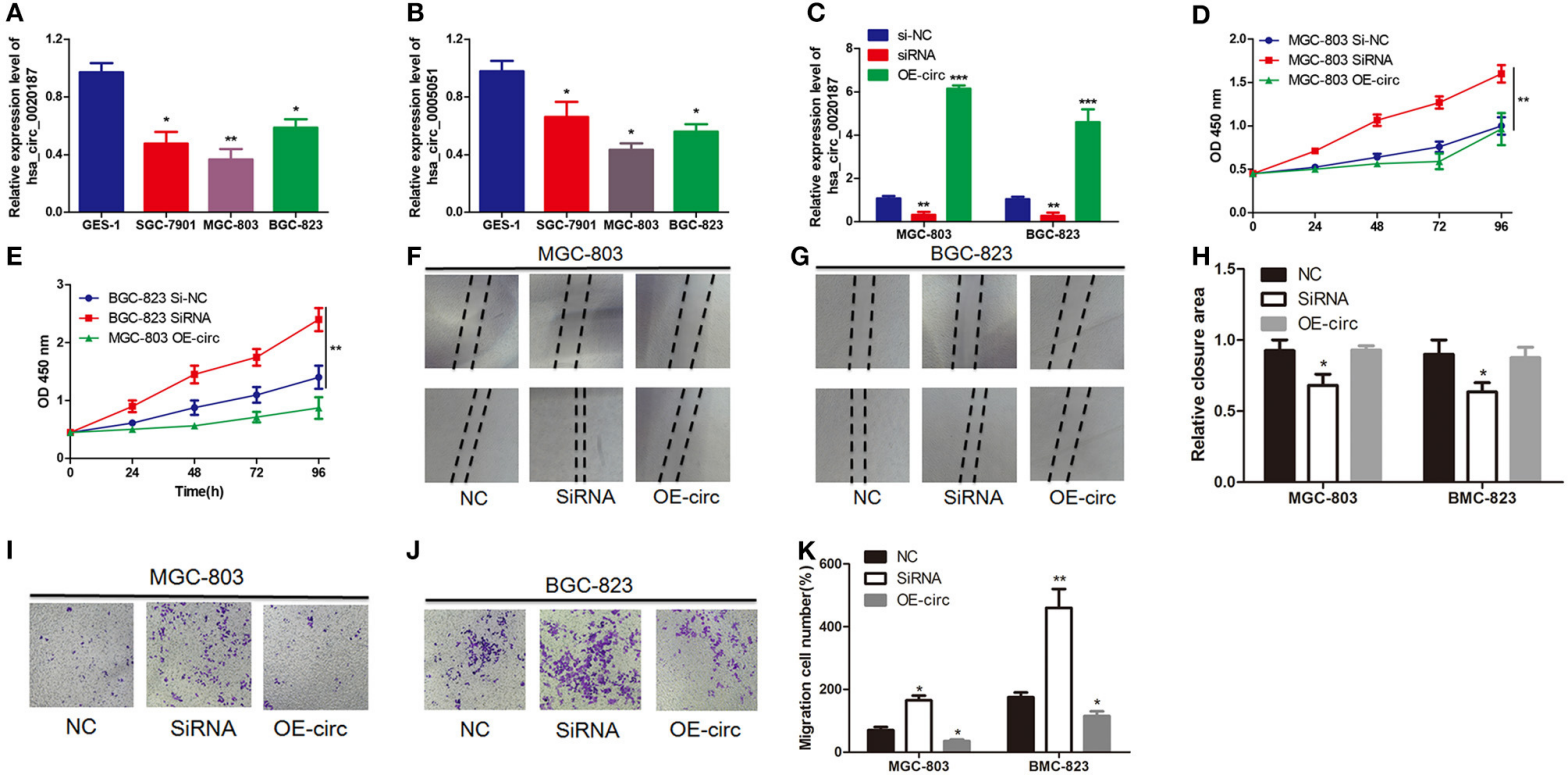
Hsa_circ_0021087 promotes GC cell proliferation *in vitro* experiments. **(A)** Hsa_circ_0021087 expression level was downregulated in GC cells (SGC-7901, MGC-803 and BGC-823) compared with the normal cell GES1. **(B)** Hsa_circ_0005051 was also downregulated in the GC cell lines. **(C)** Following the transfection of MGC-803 and BGC-823 cells with specifically synthesized small interference RNA and OE-hsa_circ_0021087, the expression of hsa_circ_0021087 in cells was analyzed by reverse transcription-quantitative PCR. **(D,E)** The Cell Counting Kit-8 assay indicated that hsa_circ_0021087 promoted MGC-803 and BGC-823 cell proliferation. **(F–H)** Wound healing assays were performed on MGC-803 and BGC-823 cells treated with si-NC, si-circ_0021087 and OE-circ_0021087. **(I–K)** Transwell chambers were used to perform cell migration assays in MGC-803 and BGC-823 cells. *P < 0.05, **P < 0.01, and ***P < 0.001. GC, gastric cancer.

Furthermore, in order to explore the roles of circRNAs in GC, preliminary experiments were carried out *in vitro*. Firstly, the expression levels of hsa_circ_0021087 and hsa_circ_0005051 were examined in the two GC cell lines. As Figures 5A,B showed that compared with human GES1, hsa_circ_0005051 in GC cell line was significantly down-regulated. Furthermore, as hsa_circ_0021087 was significantly downregulated in the blood and cells of GC, it could be used as a target to study the role of circRNA in the carcinogenesis of GC. A negative control siRNA (si-NC) or si-hsa_circ_0021087 were transfected into BGC-823 and MGC-803 cells. The RT-qPCR assay showed that siRNA downregulated the expression of hsa_circ_0021087 in GC cells compared with cells treated with si-NC and OE-circ (Figure 5C). CCK8 assay was performed to assess whether hsa_circ_0021087 was associated with cell proliferation. It was observed that proliferation was notably changed in BGC-823 and MGC-803 cells, following the expression of hsa_circ_0021087 (Figures 5D,E). These experiments indicated that hsa_circ_0021087 could promote GC cell proliferation *in vitro*. Functionally, compared with the control, cells with hsa_circ_0020187 depletion showed accelerated wound healing (Figures 5F–H). Consistently, transwell migration assays demonstrated that the inhibition of hsa_circ_0021087 led to increasing cell migration in MGC-803 and BGC-823 cells (Figures 5I–K).

### Construction the hsa_circ_0021087 of ceRNA Network

**Figure 6:**
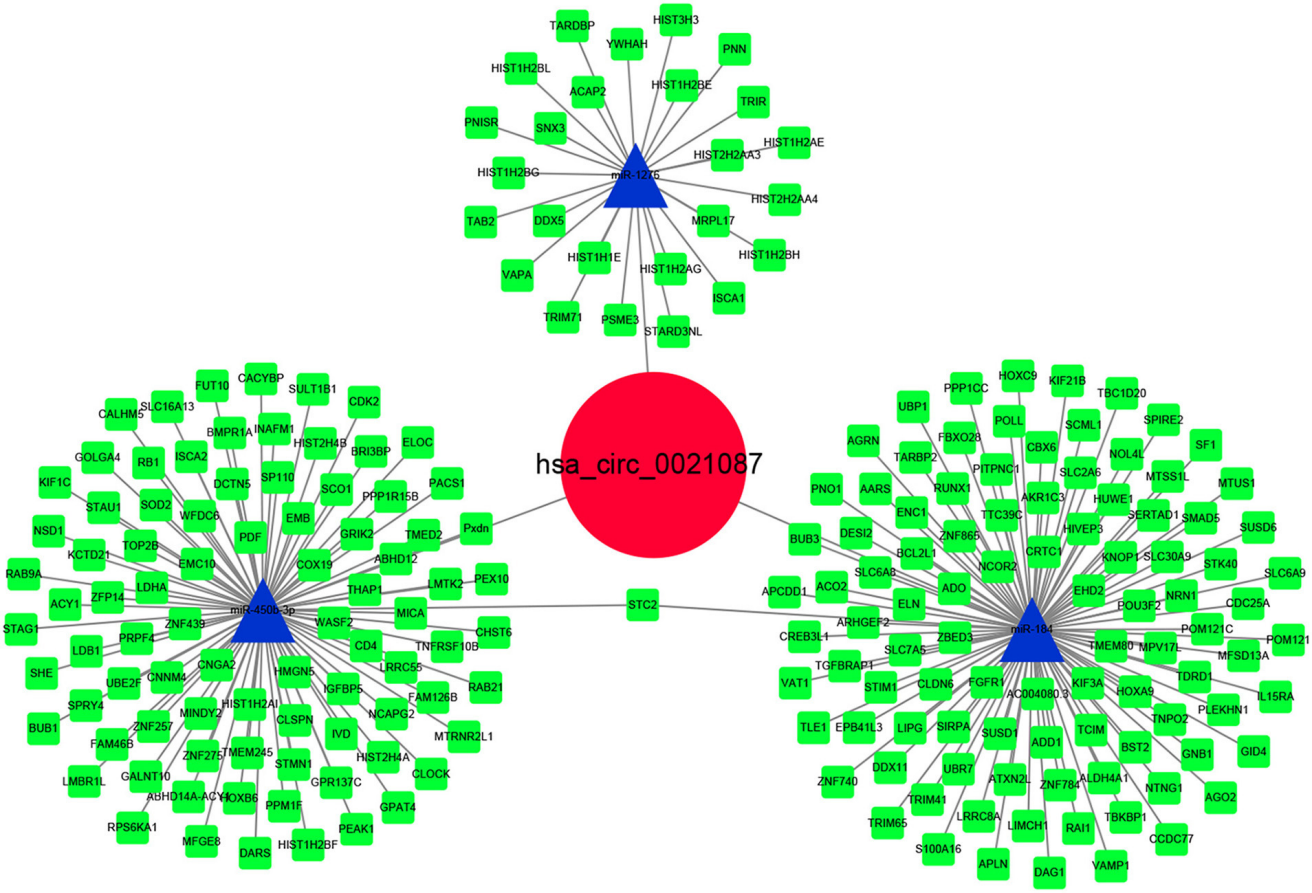
Hsa_circ_0021087–microRNA–mRNA interactions were constructed and visualized by Cytoscape software.

The CircInteractome and circBank predicted three miRNAs (hsa-miR-184, hsa-miR-1276, hsa-miR-450b-3p) after intersection that were targeted by hsa_circ_0021087 (Figure S2). Next, the target mRNAs of these miRNAs were predicted using the StarBase and TargetScan databases. Pearson correlation analysis and hypergeometric test were used to assess the ceRNA network. Finally, the interaction of three miRNAs and 211 target mRNAs are indicated in Figure 6. Additionally, the ceRNA network regulated by hsa_circ_0005051 is also illustrated in Figure S3.

### Functional Characterization of the hsa_circ_0021087 Target Genes

**Figure 7:**
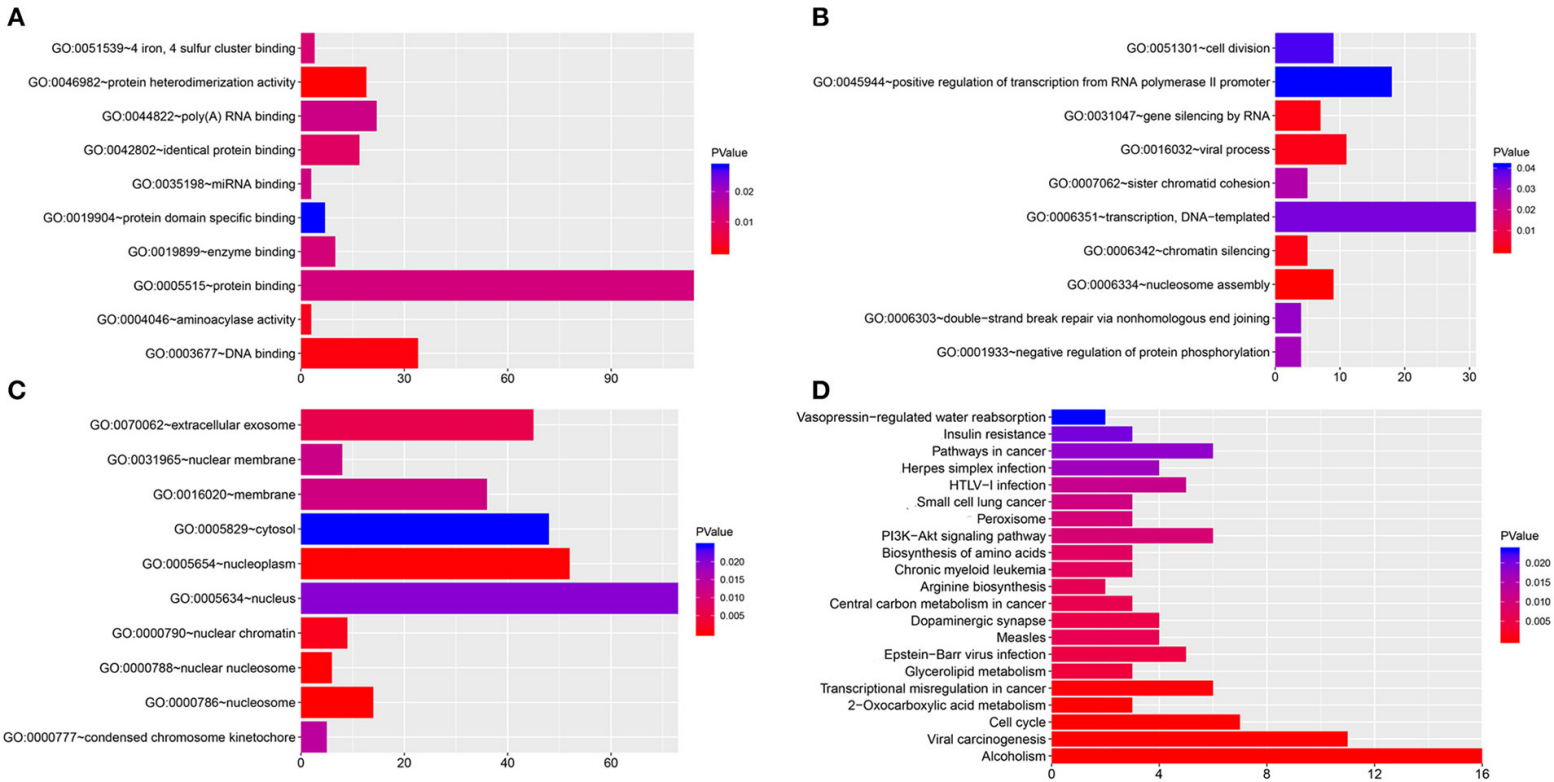
Functional analysis of hsa_circ_0021087. **(A–C)** GO analysis of hsa_circ_0021087 based on the ceRNA network. The MFs **(A)**, BPs **(B)** and CCs **(C)** of top 10 significantly enriched are listed. **(D)** KEGG pathway analysis of hsa_circ_0021087 based on the ceRNA network. GO, Gene Ontology. BPs, biological processes. MFs, molecular functions. CCs, cellular components; KEGG, Kyoto Encyclopedia of Genes and Genomes; ceRNA, competing endogenous RNA.

GO analysis was performed for hsa_circ_0021087 regulatory target genes. The top ten significantly enriched molecular functions (MFs) and biological processes (BPs), cellular components (CCs) are visualized in Figures 7A–C. In the BPs ontology, transcription, DNA-templated and positive regulation of transcription from RNA polymerase II promoter were the most enriched terms. In MFs ontology, the most enriched terms were catalytic activities and binding. In CCs ontology, nucleosome, nucleoplasm and extracellular exosome were the most enriched terms. The genes in the KEGG pathway database were also mapped, which showed that the top five most annotated genes were enriched in alcoholism, systemic lupus erythematosus, viral carcinogenesis, pathways in cancer and cell cycle (Figure 7D). In addition, the functional analysis of hsa_circ_0005051 is shown in Figure S4.

## Discussion

GC still has difficulty with early diagnosis among malignant tumors worldwide. Because of its late clinical manifestations and not enough sensitivity to radiotherapy and chemotherapy, the overall survival rate of patients with gastric cancer is still unsatisfactory. Therefore, it is very important to increase the sensitivity of early diagnosis, to develop effective prognostic biomarkers and to find new targets for GC molecular therapy.

CircRNAs have gained increasing attention for candidate biomarkers detection in the past years, due to their abundance and stability in body fluids, such as exosomes, plasma, and saliva (11, 23, 24). Several studies have published interesting results that reveal the biogenesis of circRNAs and its possible mechanisms (3, 12). In the present study, three GEO datasets were analyzed to screen for DEcircRNAs in GC, as candidate circRNAs, which was differentially expressed in TCGA-STAD data and was correlated with prognosis. It is speculated that these candidate DEcircRNAs may be associated with the pathogenesis of GC.

Subsequently, the candidate circRNAs expression levels were verified in tissues and the plasma of patients with GC. As far as we know, this is the first research to report the expression of hsa_circ_0021087 and hsa_circ_0005051 in plasma of cancer patients. Because of the advantages of availability and non-invasive, plasma samples were used in this research. Zhang et al. (25) demonstrated a difference in the expression of plasma ciRS-133 levels between GC patients and healthy controls. Furthermore, Tang et al. (13) showed lower expression of circ-KIAA1244 in GC tissues, cells and plasma compared with normal controls. The present research examined the expression level of both hsa_circ_0021087 and hsa_circ_0005051 to be differentially expressed in plasma of GC patients, and that the combination of these two types of circRNA can improve the diagnostic accuracy of GC. Additionally, the other three candidate circRNAs displayed no significant difference in the plasma of GC samples and normal samples, but this difference was attributed to the detecting method (RT-qPCR and microarray analysis), sample type (tissue and plasma) and sample size. These findings revealed that the dual circular RNA signature can be applied as a potential non-invasive biomarker for the diagnosis of GC.

Li et al. (26) reported that a number of differentially expressed circRNAs were detected in plasma of patients with cervical cancer before and after operation, some of which were considered to be prognostic biomarkers, suggesting that these circRNAs in plasma may be involved in tumor progression. Li et al. (27) demonstrated that the expression levels of hsa_circ_0061276 and hsa_circ_0001017 before and after operation in plasma can be used as independent monitoring indexes for the recurrence of GC. In this research, the expression of a specific circRNA, hsa_circ_0021087, was lower in preoperative patients with GC compared with postoperative patients, demonstrating that it may be involved in the progression and occurrence of GC. This increase may be due to a decrease in the release of nucleic acids from tumor sources that inhibit the circRNA following tumor removal, leading to convincing changes in plasma hsa_circ_0021087 levels at the preoperative and postoperative stages. It was reported that the increase of circRNA abundance in exosome may be a possible mechanism for the increase in circRNA expression (3). Yet, there was no significantly different expression of hsa_circ_0005051 before and after surgery. Therefore, this result could be attributed to the fact that this circRNA may not be increased through exosomes.

It was reported that circRNA regulates mRNA expression through competing for miRNA (3). For example, circPDSS1 can sponge miR-186-5p to promote GC cell cycle and inhibit apoptosis (28). The present study demonstrated that hsa_circ_0021087 might be associated with cell proliferation in GC, determined by the CCK-8 assay. In order to further explore this mechanism, the hsa_circ_0021087-miRNA-mRNA network was predicted and analyzed the functional enrichment of the target genes. Hence, three kinds of miRNA (miR-1286, miR-184, and miR-450b-3p) and their 205 target genes were identified. Previous findings indicated that miR-184 can inhibit the expression of SND1, MMP-2/9, CD44 in breast cancer and glioma, and act as a tumor inhibitor miRNA by inhibiting the expression of p53 and p21 and activity of caspase-3/8 in glioblastomas (29). Additionally, it was reported that hsa-miR-184 can regulate apoptosis and invasion of GC (30). A previous study has shown that miR-450b-3p was downregulated in breast cancer and could inhibit HER3 expression through the PI3K/Akt pathway (31). LIM domain only 1 (LMO1), which as a hsa_circ_0021087 host gene, has been reported as an oncogene in multiple tumors, such as lung cancer (32), neuroblastoma (33) and gastric cancer (34). Pathway analysis revealed that it is also involved in several cancer-associated pathways, such as cell cycle, viral carcinogenesis and pathways in cancer. Above results revealed that hsa_circ_0021087 may promote the development of GC as an miRNA sponge, and its mechanism is worthy of further study.

Finally, the term “circRNAs” was used on the https://clinicaltrials.gov/ online website to search for clinical trials, in order to better understand the clinical use of circRNAs. CircRNAs were recruited in clinical trials as a biomarker of acute myocardial infarction (NCT03170830) in the year 2017 and neurological outcome after cardiac arrest (NCT02297776) in the year 2014. However, there are no clinical trials on circRNA biomarkers for GC. The current study provides evidence for circulating circRNAs as a non-invasive biomarker of GC.

## Conclusion

This research first demonstrated significant downregulation of hsa_circ_0021087 and hsa_circ_0005051 in GC tissues, cells and plasma, indicating that the dual-circular RNA signature can be applied as a novel non-invasive biomarker in the diagnosis of GC. Next, hsa_circ_0021087 was demonstrated to have different expression in the plasma of GC patients at preoperative and postoperative stages and suggested that it might be involved in the occurrence and development of GC *in vitro* experiments.

## Data Availability Statement

Publicly available datasets were analyzed in this study. This data can be found here: Data is available at NCBI GEO, accession numbers: GSE83521, GSE89143, and GSE93541 and tcga database https://cancergenome.nih.gov/.

## Ethics Statement

The studies involving human participants were reviewed and approved by The Institutional Review Board of Affiliated Hospital of Qingdao University. The patients/participants provided their written informed consent to participate in this study.

## Consent for Publication

We have obtained consents to publish this paper from all the participants of this study.

## Author Contributions

LH, HH, and LY conceived and designed the experiments. LH, XZ, AW, YJ, XC, QQ, TY, LY, and HH collected and analyzed data. LH and HH wrote this manuscript. All authors read and approved the final manuscript.

### Conflict of Interest

The authors declare that the research was conducted in the absence of any commercial or financial relationships that could be construed as a potential conflict of interest.

## Supplementary Material

Table S1 Detailed clinical information for QRT-PCR detection of circRNAs.

Table S2 Primers of circRNAs.

Figure S1 Hsa_circ_0021087 is derived from the second and third exons of the LIM domain only 1 gene on chromosome 21, and hsa_circ_0005051 is produced at the UBXN7 gene locus containing exon 4, 5 on chromosome 3.

Figure S2 The number of circular RNAs that can interact with microRNAs comes from the intersection of the circBank and circinteractome databases.

Figure S3 Hsa_circ_0005051–microRNA–mRNA interactions were constructed and visualized by Cytoscape software.

Figure S4 Functional analysis of hsa_circ_0005051. **(a)** GO analysis of hsa_circ_0005051 based on the ceRNA network. The BPs, MFs and CCs of top ten significantly enriched are listed. **(b)** KEGG pathway analysis of hsa_circ_0005051 based on the ceRNA network. GO, Gene Ontology. BPs, biological processes. MFs, molecular functions. CCs, cellular components. KEGG, Kyoto Encyclopedia of Genes and Genomes. ceRNA, competing endogenous RNA.
