# Corrigendum: A Dual-Circular RNA Signature as a Non-invasive Diagnostic Biomarker for Gastric Cancer

**DOI:** 10.3389/fonc.2020.01704

**Published:** 2020-09-18

**Authors:** Li Han, Xiaoying Zhang, Aimin Wang, Yang Ji, Xuelei Cao, Qiaoji Qin, Tao Yu, Huan Huang, Lei Yin

**Affiliations:** ^1^School of Nursing, Qingdao University, Qingdao, China; ^2^Department of Cardiology, The Affiliated Hospital of Qingdao University, Qingdao, China; ^3^Department of Emergency Internal Medicine, The Affiliated Hospital of Qingdao University, Qingdao, China

**Keywords:** gastric cancer, circRNAs, plasma, diagnosis, signature, Hsa_circ_0020187, Hsa_circ_0005051

In the original article the author order was incorrectly given as **Lei Yin and Huan Huang**. The correct order is **Huan Huang and Lei Yin**.

The authors apologize for this error and state that this does not change the scientific conclusions of the article in any way. The original article has been updated.

