# Supplementary material for: A Dual-Circular RNA Signature as a Non-invasive Diagnostic Biomarker for Gastric Cancer
Source: Front Oncol. 2020 Feb 21;10:184. doi: 10.3389/fonc.2020.00184 (PMC7047344; doi:10.3389/fonc.2020.00184)
Supplement: Table S1 — Detailed clinical information for QRT-PCR detection of circRNAs. [file Table_1.DOCX]

Table S1 Detailed clinical information for QRT-PCR detection of circRNAs

| Number | Time | Gender | Age | Pathology | Tumor size(cm) | Tumor location | Stage |
| --- | --- | --- | --- | --- | --- | --- | --- |
| 1 | 20180306 | Male | 78 | Adenocarcinoma | 4.5×3×1.2 | Lesser curvature | IIA |
| 2 | 20180306 | Female | 70 | Adenocarcinoma | 3.5×2.5×1.2 | Gastric body | IIB |
| 3 | 20180309 | Male | 59 | Adenocarcinoma | 6×5.5×1.5 | Cardia | IIIA |
| 4 | 20180309 | Female | 53 | Adenocarcinoma | 3.5×2.5×1.2 | Antrum | IIA |
| 5 | 20180313 | Female | 66 | Adenocarcinoma | 8×7×2 | Gastric body | IIIA |
| 6 | 20180313 | Male | 66 | Adenocarcinoma | 5.5×2.5×1.5 | Cardia | IIB |
| 7 | 20180313 | Male | 59 | Adenocarcinoma | 6×5.5×1 | Gastric body | IIB |
| 8 | 20180313 | Male | 63 | Adenocarcinoma | 4.5×3.5×1.3 | Gastric angle | IIIB |
| 9 | 20180316 | Male | 87 | Adenocarcinoma | 2.5×1.8×1 | Gastric body | I |
| 10 | 20180319 | Female | 64 | Adenocarcinoma | 4.5×3×1.5 | Lesser curvature | IIB |
| 11 | 20180319 | Male | 79 | Adenocarcinoma | 6×4×1.8 | GNor | IIIA |
| 12 | 20180319 | Male | 53 | Adenocarcinoma | 3×3×1.2 | GNor | I |
| 13 | 20180323 | Male | 59 | Adenocarcinoma | 4.5×3.5×1.3 | Gastric body | IIIB |
| 14 | 20180323 | Male | 57 | Adenocarcinoma | 4.5×3×1.5 | GNor | IIIB |
| 15 | 20180323 | Male | 62 | Adenocarcinoma | 3×2.7×1 | Gastric body | IIA |
| 16 | 20180323 | Male | 65 | Adenocarcinoma | 4×3.5×1.6 | GNor | IIIB |
| 17 | 20180323 | Female | 67 | Adenocarcinoma | 4×3.5×2 | Cardia | IIIA |
| 18 | 20180326 | Female | 62 | Adenocarcinoma | 4.5×3×1.5 | GNor | IIIA |
| 19 | 20180330 | Male | 81 | Adenocarcinoma | 6×4×1.8 | GNor | IIIB |
| 20 | 20180330 | Male | 74 | Adenocarcinoma | 4×3.5×2 | Cardia | IIIB |
| 21 | 20180402 | Male | 55 | Adenocarcinoma | 4.5×3.5×1.3 | Gastric angle | IIB |
| 22 | 20180402 | Female | 65 | Adenocarcinoma | 4×2×1.2 | GNor | IIB |
| 23 | 20180402 | Female | 62 | Adenocarcinoma | 3×2.7×1 | Antrum | IIB |
| 24 | 20180402 | Male | 53 | Adenocarcinoma | 8×5×2 | Lesser curvature | IIIA |
| 25 | 20180406 | Male | 83 | Adenocarcinoma | 6×4.5×2 | Corpus | IIIB |
| 26 | 20180406 | Male | 55 | Adenocarcinoma | 3.5×2.5×2 | Gastric body | IIIA |
| 27 | 20180406 | Male | 77 | Adenocarcinoma | 4.5×3×2 | GNor | IIB |
| 28 | 20180409 | Female | 67 | Adenocarcinoma | 6.5×5×1.8 | Gastric body | IIIB |
| 29 | 20180413 | Female | 49 | Adenocarcinoma | 8×5×2.9 | GNor | IIIA |
| 30 | 20180413 | Male | 74 | Adenocarcinoma | 6.5×4×3.5 | Gastric body | IIIB |
| 31 | 20180413 | Male | 49 | Adenocarcinoma | 4.5×3.5×1.2 | Gastric angle | IIB |
| 32 | 20180416 | Female | 74 | Adenocarcinoma | 12×8×2 | Gastric body | IIIB |
| 33 | 20180416 | Female | 49 | Adenocarcinoma | 3.5×2.5×1.3 | Corpus | IIA |
| 34 | 20180420 | Male | 65 | Adenocarcinoma | 3×2×1 | GNor | I |
| 35 | 20180420 | Female | 62 | Adenocarcinoma | 8×4.5×1.6 | GNor | IIIB |
| 36 | 20180423 | Female | 81 | Adenocarcinoma | 4.5×3×1.5 | GNor | IIA |
| 37 | 20180423 | Male | 64 | Adenocarcinoma | 4×2×1.2 | Gastric body | IIB |
| 38 | 20180423 | Male | 68 | Adenocarcinoma | 4.5×3×1.2 | Antrum | IIIB |
| 39 | 20180423 | Female | 85 | Adenocarcinoma | 3.5×2.5×1.2 | Lesser curvature | IIB |
| 40 | 20180427 | Female | 82 | Adenocarcinoma | 4.5×3×1.5 | Gastric body | IIB |
| 41 | 20180430 | Male | 65 | Adenocarcinoma | 6×4×1.8 | Lesser curvature | IIIB |
| 42 | 20180430 | Male | 87 | Adenocarcinoma | 3×3×1.8 | Gastric body | IIA |
| 43 | 20180407 | Male | 69 | Adenocarcinoma | 6×3×1.2 | Gastric angle | IIIB |
| 44 | 20180407 | Male | 62 | Adenocarcinoma | 5×3.5×1.8 | Gastric angle | IIIB |
| 45 | 20180407 | Male | 51 | Adenocarcinoma | 9×8×2.6 | Corpus | IIIA |
| 46 | 20180407 | Female | 58 | Adenocarcinoma | 8×4×2.2 | Gastric body | IIIB |
| 47 | 20180511 | Male | 83 | Adenocarcinoma | 6.3×5.5×2.5 | Antrum | IIIB |
| 48 | 20180514 | Male | 82 | Adenocarcinoma | 5.5×5.5×2 | Gastric angle | IIB |
| 49 | 20180514 | Male | 55 | Adenocarcinoma | 3.5×2.5×1.2 | GNor | IIA |
| 50 | 20180514 | Female | 69 | Adenocarcinoma | 4.5×3×1.5 | GNor | IIB |
| 51 | 20180514 | Female | 74 | Adenocarcinoma | 6×3×2 | Lesser curvature | IIIB |
| 52 | 20180518 | Male | 65 | Adenocarcinoma | 7×5×3 | Gastric body | IIIA |
| 53 | 20180518 | Male | 65 | Adenocarcinoma | 7×5.5×4 | Gastric body | IIIB |
| 54 | 20180521 | Male | 57 | Adenocarcinoma | 10.8×9×2 | GNor | IIIA |
| 55 | 20180521 | Female | 63 | Adenocarcinoma | 3.5×2.5×2 | Gastric body | IIB |
| 56 | 20180521 | Female | 52 | Adenocarcinoma | 3×2.7×1 | Gastric angle | IIB |
| 57 | 20180521 | Male | 36 | Adenocarcinoma | 3.5×2.5×1.2 | Antrum | IIB |
| 58 | 20180525 | Female | 77 | Adenocarcinoma | 6.5×5×1.8 | GNor | IIA |
| 59 | 20180528 | Female | 64 | Adenocarcinoma | 3.5×2.5×1.2 | Lesser curvature | IIA |
| 60 | 20180528 | Male | 51 | Adenocarcinoma | 6.5×4×3.5 | Corpus | IIIB |
| 61 | 20180601 | Female | 66 | Adenocarcinoma | 8×4×2.5 | Corpus | IIIB |
| 62 | 20180601 | Male | 73 | Adenocarcinoma | 4.5×3×1.5 | Lesser curvature | IIA |
| 63 | 20180601 | Male | 52 | Adenocarcinoma | 6.5×5×4 | Gastric body | IIIA |
| 64 | 20180604 | Male | 59 | Adenocarcinoma | 6×4.5×2 | Gastric body | IIIA |
| 65 | 20180604 | Female | 56 | Adenocarcinoma | 3.5×2.5×2 | GNor | IIA |
| 66 | 20180604 | Female | 65 | Adenocarcinoma | 4×2×1.2 | Lesser curvature | I |
| 67 | 20180608 | Male | 59 | Adenocarcinoma | 6×3×2 | Antrum | IIIB |
| 68 | 20180608 | Male | 69 | Adenocarcinoma | 5.5×2.5×1.5 | Gastric angle | IIB |
| 69 | 20180611 | Female | 76 | Adenocarcinoma | 12×8×2 | Gastric body | IIIA |
| 70 | 20180615 | Male | 65 | Adenocarcinoma | 4.5×3×1.2 | GNor | IIIB |
