# Supplementary material for: A Dual-Circular RNA Signature as a Non-invasive Diagnostic Biomarker for Gastric Cancer
Source: Front Oncol. 2020 Feb 21;10:184. doi: 10.3389/fonc.2020.00184 (PMC7047344; doi:10.3389/fonc.2020.00184)
Supplement: Table S2 — Primers of circRNAs. [file Table_2.DOCX]

Table S2 Primers of circRNAs

| Hsa_circ_0021087 | F: CTCTTTGGCACCACAGGGAACT |
| --- | --- |
|  | R: TCCAATGCCTTCAGCAGATAGC |
| Hsa_circ_0005051 | F: AAACACTCCTAAAAGACGACGG |
|  | R: ATCTGCAAGGGTAGTTAATTTCTTATC |
| Hsa_circ_0000332 | F: CTCGGGCTGTGGCCTCAC |
|  | R: AGAACTGCGGGTGCTG |
| Hsa_circ_0007518 | F: GGTGCTGGGTCACTTTCCGTAT |
|  | R: AGTCCGCTGAAGAACTCGCTCT |
| Hsa_circ_0000554 | F: TGGGTAAAAGGACAGGGGGATG |
|  | R: AGGGGCAGAGACAGAGTGGATG |
| GAPDH | F: CTGACTTCAACAGCGACACC |
|  | R: TGCTGTAGCCAAATTCGTTGT |
| Si-hsa_circ_0021087 | CGTTGGTCTCCGCACGGCTACGAGA |
| Si-hsa_circ_0005051 | GAAACTTTGTGAGGATTTTCTGCTG |
