# Supplementary figures and images for: A Dual-Circular RNA Signature as a Non-invasive Diagnostic Biomarker for Gastric Cancer
Source: Front Oncol. 2020 Feb 21;10:184. doi: 10.3389/fonc.2020.00184 (PMC7047344; doi:10.3389/fonc.2020.00184)

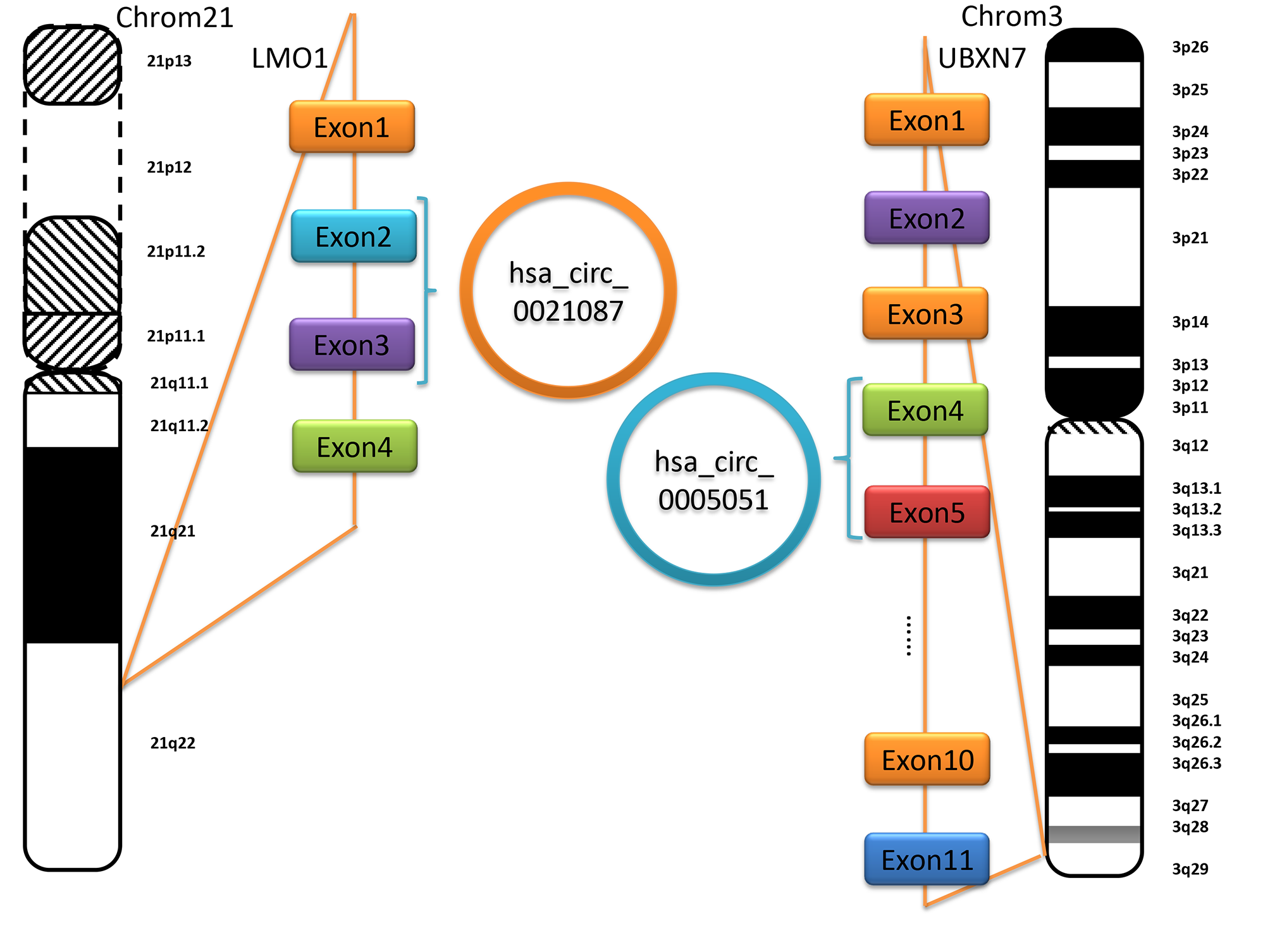

Supplement: Figure S1 — Hsa_circ_0021087 is derived from the second and third exons of the LIM domain only 1 gene on chromosome 21, and hsa_circ_0005051 is produced at the UBXN7 gene locus containing exon 4, 5 on chromosome 3. [file Image_1.TIF]

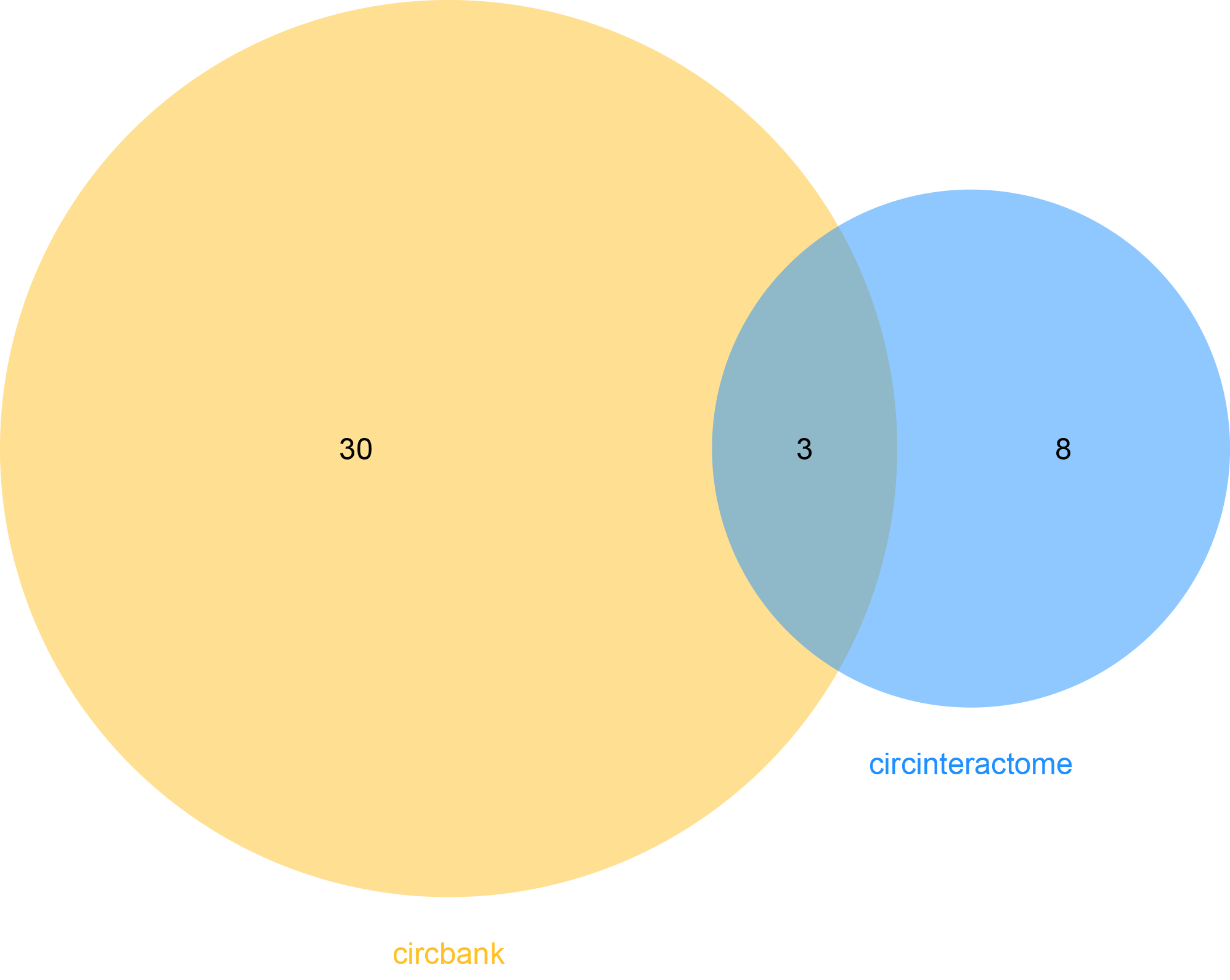

Supplement: Figure S2 — The number of circular RNAs that can interact with microRNAs comes from the intersection of the circBank and circinteractome databases. [file Image_2.TIF]

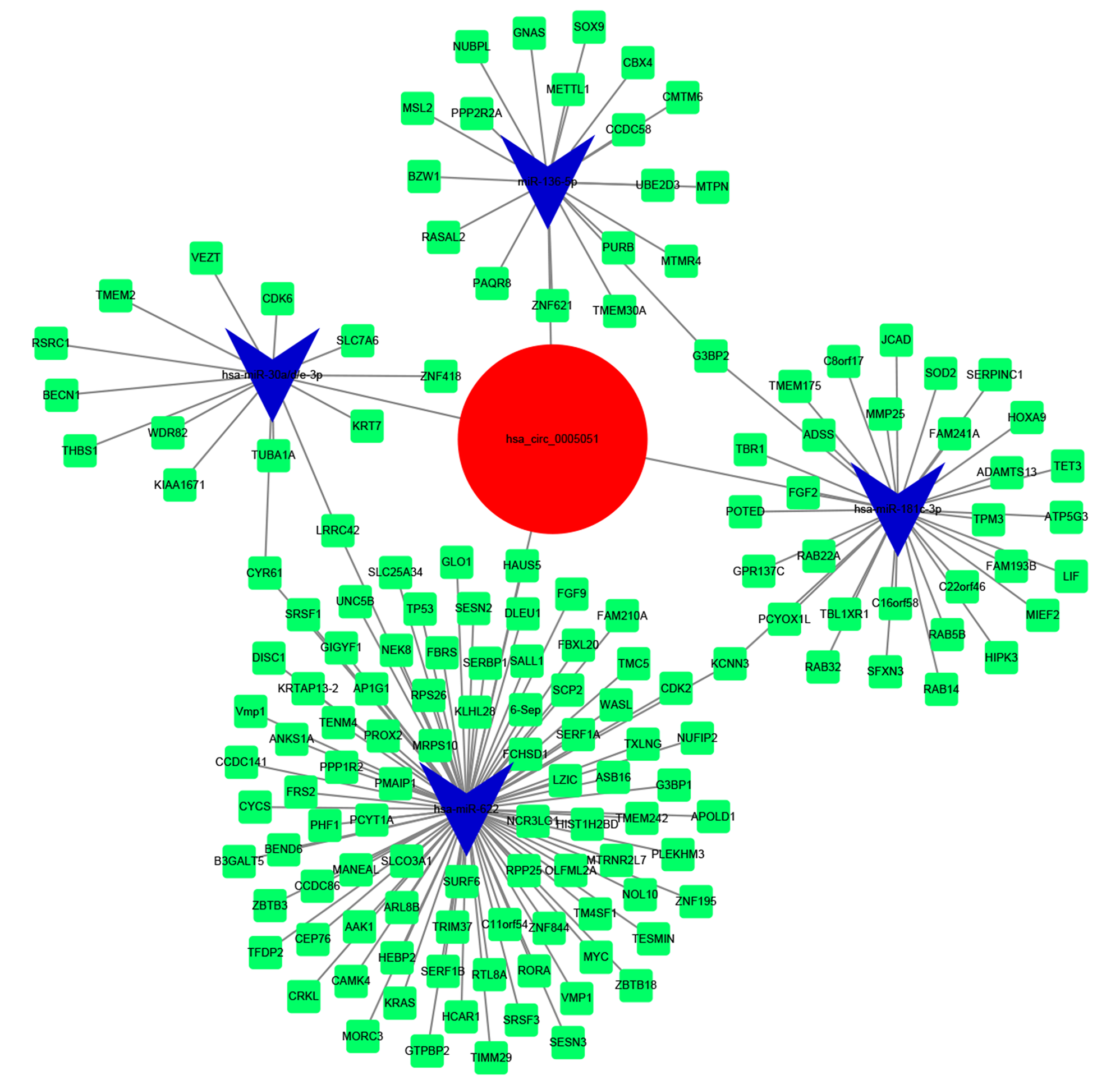

Supplement: Figure S3 — Hsa_circ_0005051–microRNA–mRNA interactions were constructed and visualized by Cytoscape software. [file Image_3.TIF]

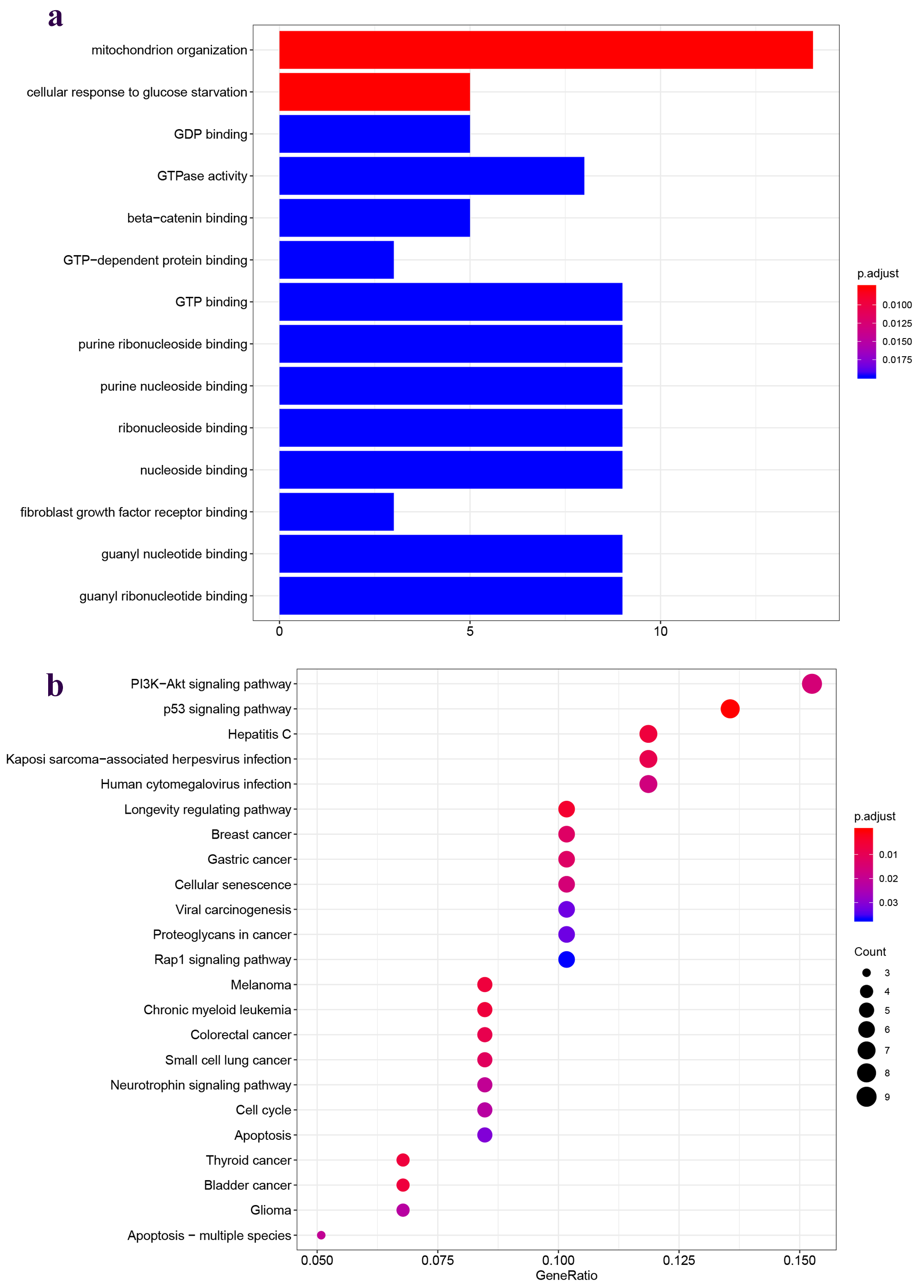

Supplement: Figure S4 — Functional analysis of hsa_circ_0005051. (a) GO analysis of hsa_circ_0005051 based on the ceRNA network. The BPs, MFs and CCs of top ten significantly enriched are listed. (b) KEGG pathway analysis of hsa_circ_0005051 based on the ceRNA network. GO, Gene Ontology. BPs, biological processes. MFs, molecular functions. CCs, cellular components. KEGG, Kyoto Encyclopedia of Genes and Genomes. ceRNA, competing endogenous RNA. [file Image_4.TIF]
